# Effect of Mechanical Heterogeneity on Creep and Stress Corrosion Cracking Propagation in Nuclear Safe-End Dissimilar Metal Welded Joints

**DOI:** 10.3390/ma19173722

**Published:** 2026-09-01

**Authors:** Jianlong Zhang, Yinghao Cui, Yongxian Chen

**Affiliations:** 1 R&D Department, Xi’an Special Equipment Inspection Institute, Xi’an 710065, China; zhangjl@chd.edu.cn; 2 School of Intelligent Mechatronics Engineering, Zhongyuan University of Technology, Zhengzhou 450007, China; 3 Chengdu Special Equipment Emergency Response Center, Chengdu Special Equipment Inspection and Testing Research Institute, Chengdu 610036, China

**Keywords:** stress corrosion cracking, dissimilar metal welded joints (DMWJs), mechanical heterogeneity, crack tip creep

## Abstract

The dissimilar metal welded joints at the safe ends of nuclear primary circuits are highly susceptible to stress corrosion cracking (SCC) initiation in high-temperature, high-pressure water environments. Existing predictive models are predominantly based on homogeneous material assumptions, making it challenging to accurately evaluate the actual failure behavior of welds caused by mechanical property heterogeneity. Consequently, based on the mechanical gradient obtained from hardness tests, this study constructs a finite element model with continuously varying mechanical properties to quantitatively investigate SCC behavior under different crack characteristics. The analysis demonstrates that mechanical heterogeneity significantly influences the crack tip mechanical fields: When the crack is located proximal to the sub-interface (d = 1 mm), the severe mechanical mismatch induces a sharp increase in creep strain, resulting in a peak SCC propagation rate approximately 14.6% higher than those at d = 3 mm. Furthermore, extending the crack length at the weld center (*a/W* from 0.45 to 0.60) expands the plastic strain zone along the propagation direction, driving an approximately 43.6% increase in the crack growth rate. The heterogeneous model, accounting for the local mechanical gradient, can more accurately reveal the influence laws of crack position and length on SCC propagation behavior, providing theoretical support for improving life prediction accuracy and in-service inspections.

## 1. Introduction

The safe ends of nuclear primary circuits typically utilize dissimilar metal welded joints (DMWJs) to connect the pressure vessel with the main pipeline. Operating in high-temperature, high-pressure, and highly corrosive aqueous environments for prolonged periods, these joints are extremely prone to the initiation of stress corrosion cracking (SCC) [1,2,3,4]. Historical operational data indicate that coolant leakage induced by SCC represents one of the primary failure modes threatening the structural integrity of nuclear power plants [5,6].

Currently, the “slip-dissolution/film rupture model” serves as the mainstream theory explaining the environment-assisted cracking process [7,8,9,10]. Numerous studies have pointed out that creep in the high-stress region at the crack tip under high-temperature and high-pressure water environments is the core driving force leading to the rupture of the passivating oxide film, thereby triggering SCC propagation [11,12,13]. However, existing SCC predictions predominantly target homogeneous materials. Due to the specificities of the welding process, significant microstructural and mechanical property heterogeneities exist within the weld and heat-affected zones of DMWJs [14,15,16,17]. For instance, Yang et al. [18] and Cui et al. [13,19] established frameworks based on crack tip creep strain rate to predict SCC growth rates in nuclear structural materials, while Ford [8] and Yi & Was [20] elaborated on the mechanistic links between creep and environmental cracking. However, these models predominantly target homogeneous materials or simplified geometries.

Due to the specificities of the welding process, significant microstructural and mechanical property heterogeneities exist within the weld and heat-affected zones of DMWJs. Such macroscopic and microscopic mechanical gradients lead to severe asymmetry and stress concentration within the stress–strain fields at the crack tip. Recent studies by Wang et al. [3,14,21] and others [4,15,22] have extensively investigated the inhomogeneous mechanical fields and crack driving forces in DMWJs, revealing how mechanical mismatch between different material zones affects local stress states. While these works provide valuable insights into the mechanical behavior, they do not fully integrate the continuous variation in local mechanical properties with quantitative SCC propagation predictions considering crack geometry parameters.

For actual weld regions exhibiting mechanical property gradients, the effects of crack initiation position and propagation length on the crack tip mechanical fields, creep fields, and SCC propagation rates remain insufficiently understood. Compared to previous studies, the novelty of the present work lies in: (i) introducing Vickers hardness testing to acquire the actual local yield strength distribution and establishing a continuous-gradient finite element model featuring continuously varying mechanical properties; (ii) combining this heterogeneous model with a quantitative prediction theory based on creep-induced oxide film rupture to systematically investigate the influence of both crack position (relative to the sub-interface) and crack length on SCC propagation rates; and (iii) revealing how mechanical mismatch at the sub-interface accelerates creep and SCC, providing a more accurate tool for life assessment of critical nuclear power components. In conjunction with a quantitative prediction theory based on creep-induced oxide film rupture, this study deeply investigates the mechanisms by which crack position and length affect crack tip fields and SCC propagation rates. This holds significant engineering value for enhancing the life assessment accuracy of critical nuclear power components.

## 2. Experimental Methods and Finite Element Model

### 2.1. Characterization and Acquisition of Mechanical Heterogeneity

**Figure 1:**
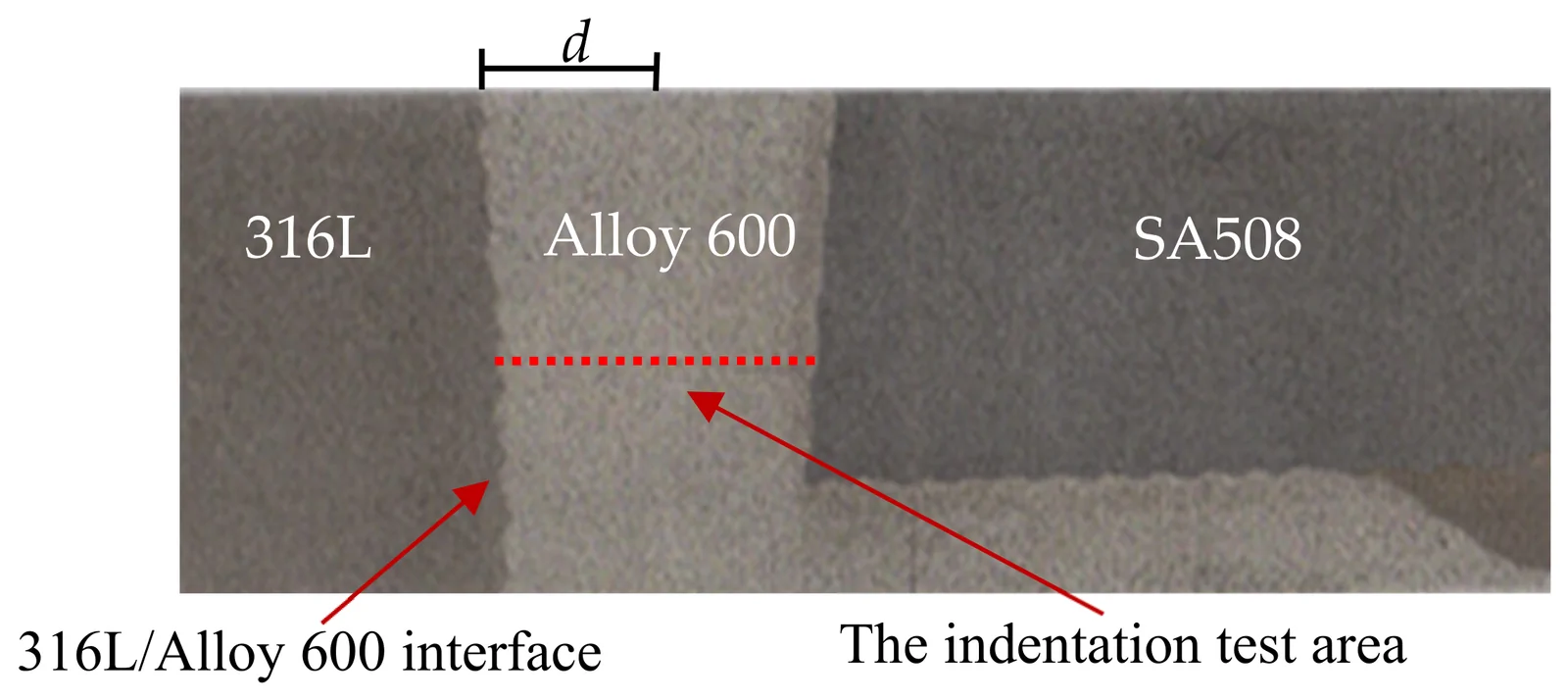
Physical image of a typical DMWJ at the nuclear pressure vessel safe end.

**Figure 2:**
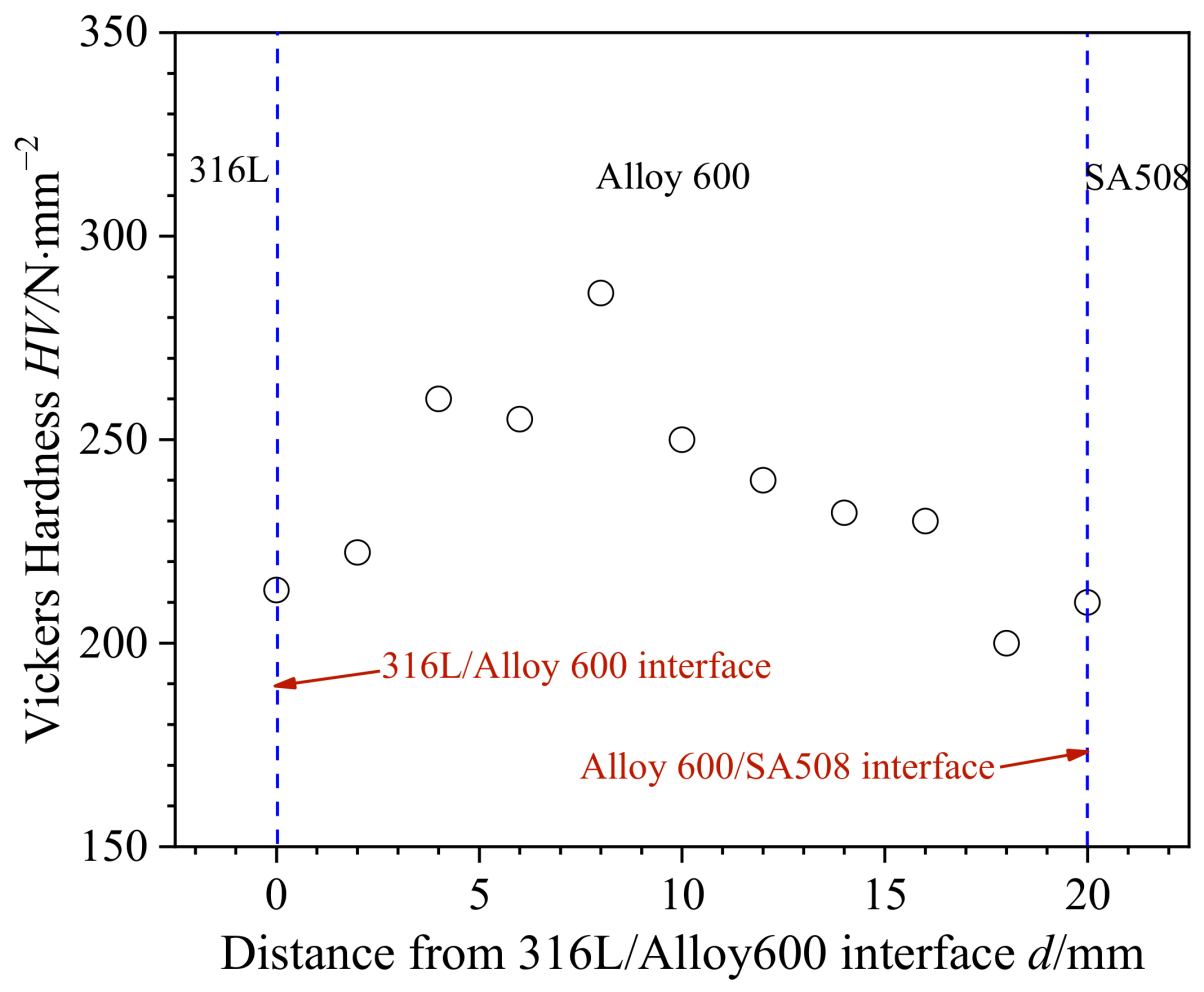
Hardness distribution in the weld region of the joint.

The research object is a typical DMWJ of a nuclear power safe end, generally composed of low-alloy steel (SA508), a nickel-based alloy weld (Alloy 600), and austenitic stainless steel (316L) [23,24,25]. Owing to the pronounced heterogeneity inherent in welded joints, conventional uniaxial tensile testing is impractical for characterizing the spatial distribution of mechanical properties. Consequently, methodologies leveraging the empirical correlation between hardness and material performance have been extensively adopted to map these property gradients [26,27,28]. Tests were performed perpendicular to the weld direction, advancing from the 316L stainless steel base metal toward the nickel-based alloy weld region. A Vickers diamond indenter was used under a load of 10 N with a 10 s dwell time. To ensure repeatability and avoid accidental errors, three independent hardness indentations were performed at each measurement location. The measurement uncertainty was assessed based on the scatter of these repeated tests. The spacing between adjacent measurement locations was set to 2 mm. Based on the conversion relationship between hardness and yield strength established by Guo [29], a continuous distribution field function of yield strength varying with distance d from the weld was obtained via polynomial fitting. A more detailed description of the testing protocol can be found in Reference [29]. The measurement region is depicted in Figure 1; the spacing between adjacent indents in the hardness testing was set to 2 mm, and the hardness measurement results for the weld region of the joint specimen are shown in Figure 2.

**Figure 3:**
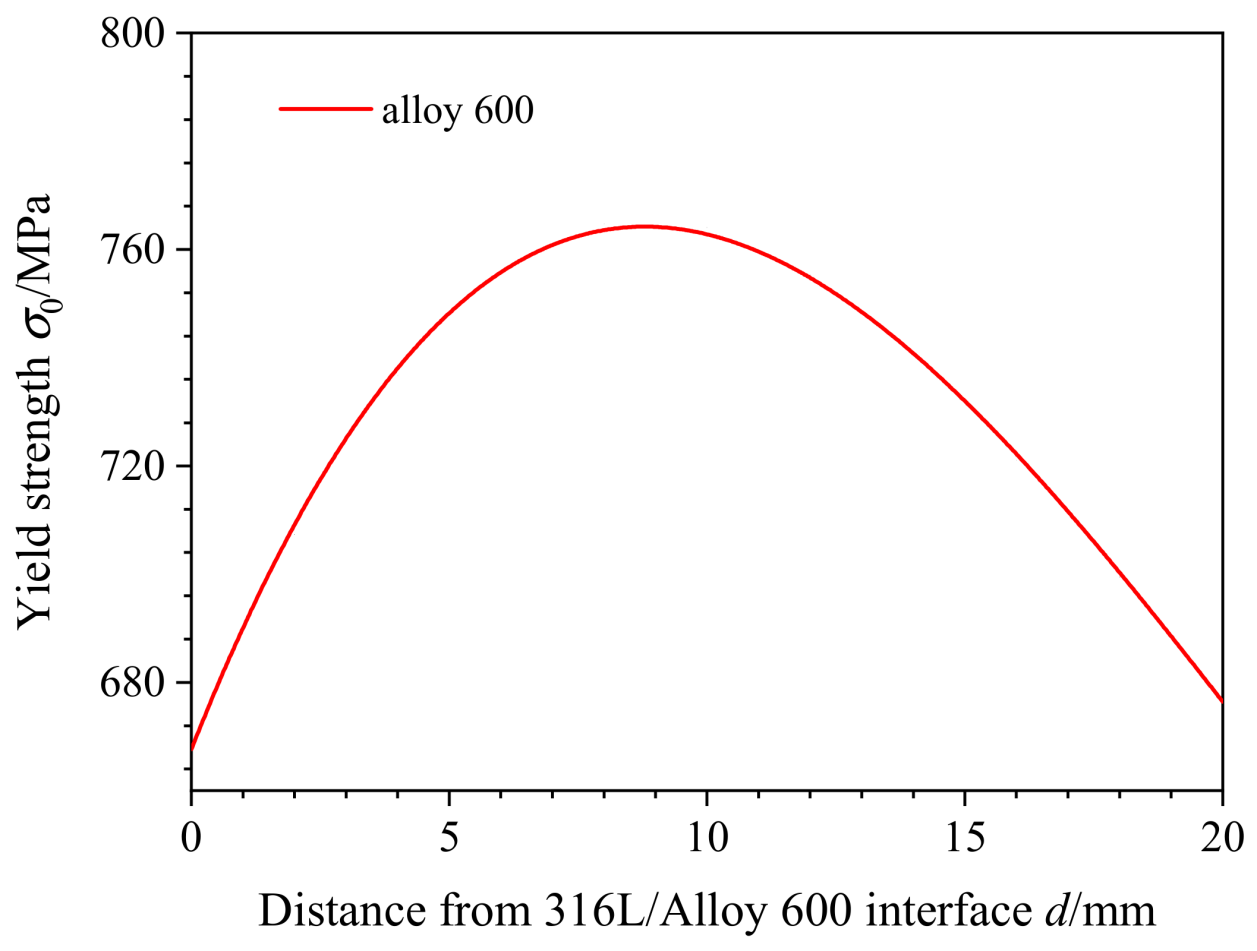
Fitted curve showing the relationship between yield strength and distance from the weld region.

The test results demonstrate that the hardness in the 316L region near the left side of the weld is relatively low, exhibiting a non-linear increasing trend as it enters the nickel-based alloy weld zone. Based on the conversion relationship between hardness and yield strength, a continuous distribution field function of yield strength varying with distance d from the weld was obtained via polynomial fitting [29]. Ultimately, a fitted curve representing the heterogeneous continuous mechanical property field in the weld region was established, as shown in Figure 3. In Figure 2 and Figure 3, *d* denotes the perpendicular distance from the 316L/Alloy 600 fusion boundary to the hardness test site.

*σ*_0_ = 667.7 + 24.1 × *d* − 1.7 × *d*^2^ + 0.0275 × *d*^3^ (1)

### 2.2. Construction of the “Sandwich” Finite Element Model

#### 2.2.1. Geometric Model

**Figure 4:**
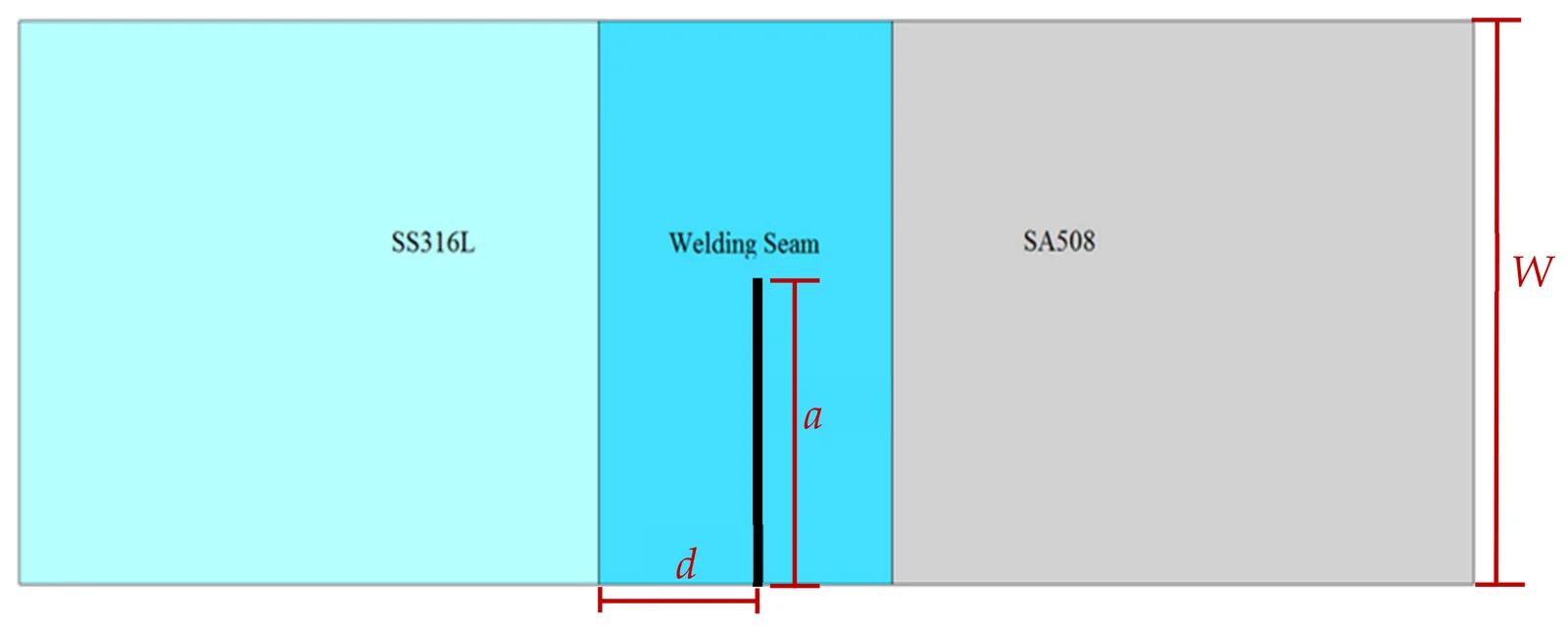
Schematic diagram of crack position and crack length in the welded joint.

Based on an actual nuclear safe end DMWJ, a “sandwich” structured finite-width plate tension model with dimensions of 120 mm × 60 mm and a weld width of 20 mm was constructed in ABAQUS, as shown in Figure 4. The materials comprise austenitic stainless steel 316L, nickel-based Alloy 600, and low-alloy steel SA508. To investigate the effects of different SCC crack positions and lengths on the crack tip mechanical fields and propagation rates, crack positions located at the 316L-Alloy 600 sub-interface on the near-weld side were set to *d* = 1 mm, 3 mm, and 5 mm. Additionally, varying crack propagation lengths *a/W* at the weld center were sequentially set to 0.45, 0.5, 0.55, and 0.6.

#### 2.2.2. Material Mechanical Model and Boundary Conditions

The stress–strain mechanical property curves of the low-alloy steel A508 and austenitic stainless steel 316L base metals can be obtained through tensile testing [30]. The yield strength of low-alloy steel A508 is 512 MPa, while that of stainless steel 316L is 345 MPa. For the core weld zone, this study utilized the predefined temperature field function in ABAQUS 6.14 to map the spatial function of Equation (1) into the model, thereby realizing a continuous spatial distribution of the mechanical property gradient. One end of the model was fixed, and a uniform tensile load was applied to the opposite end to ensure the initial stress intensity factor reached *K* = 15 MPa∙m^0.5^. The applied stress intensity factor of *K* = 15 MPa·m^1/2^ was carefully selected based on values widely adopted in the literature for similar nuclear-grade DMWJs operating in high-temperature pressurized water environments [31,32]. The creep analysis step duration was set to 1000 h.

#### 2.2.3. Mesh Model

**Figure 5:**
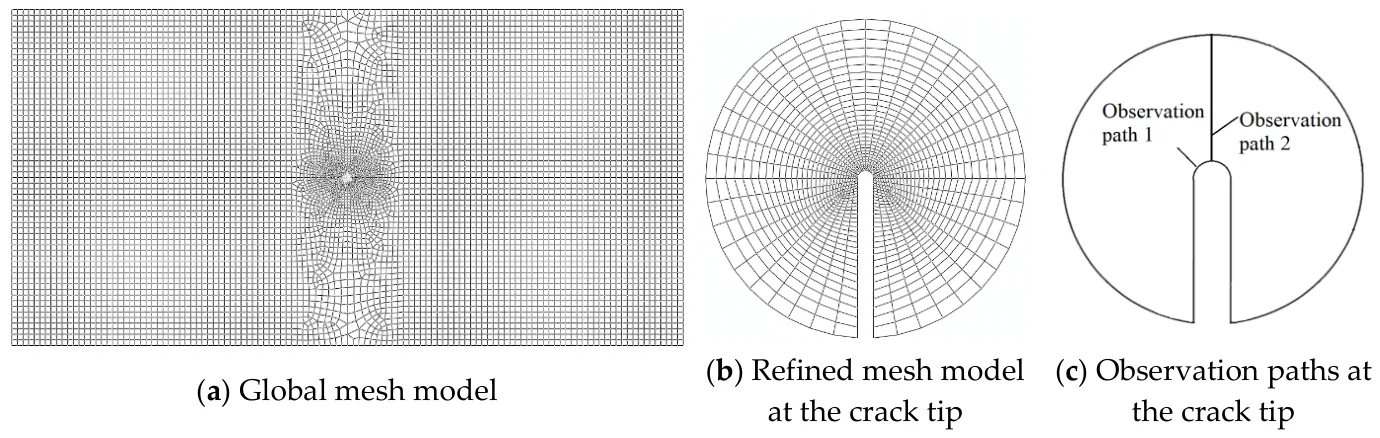
Finite element mesh model and schematic of observation paths for the welded joint.

Considering that the actual welded joint experiences full constraint in the thickness direction, equating to a plane strain state, a two-dimensional plane strain element (CPE4) was employed. To precisely capture the high-gradient strains at the crack tip, a sub-modeling technique was applied to significantly refine the crack tip region [21,33]. Simultaneously, to clearly characterize the variation laws of stress, strain, and creep at the crack tip, observation paths 1 and 2 were established along the crack propagation direction and the crack tip circumferential direction, respectively, as illustrated in Figure 5.

### 2.3. Theory of SCC Propagation Rate Prediction Based on Creep-Induced Film Rupture

This study employs a theoretical framework based on creep-induced oxide film rupture to predict the SCC propagation rate. The theory postulates that the accumulation of high-stress steady-state creep strain in the base material at the crack tip is the primary cause of surface oxide film rupture. Combined with Faraday’s law and the slip-dissolution mechanism, the SCC propagation rate can be quantitatively expressed as Equation (2).

(2) $$\frac{da}{dt}=\frac{M}{Z\cdot \rho \cdot F}\times \frac{i_{0}}{1-m}\times {\left(\frac{t_{0}}{\varepsilon_{f}}\right)}^{m}\times {\left(\varepsilon_{cr}^{\prime }\right)}^{m}$$

where *M* is the atomic weight; *Z* is the charge change during dissolution; *i*_0_ is the current density of crack tip oxidation; *ε_f_* is the fracture strain of the oxide film on the crack tip surface; *ρ* is the density; *m* is the current decay curve exponent; *F* is the Faraday constant; *t*_0_ is the time constant (s); and $\varepsilon_{cr}^{\prime }$ is the steady-state creep rate at the crack tip. The creep behavior conforms to a power-law model $\varepsilon_{cr}^{\prime }=A\times \sigma^{q}$ [20], The relevant creep parameters—the power-law multiplier *A* = 7.08 × 10^−16^ MPa^−*q*^ × *h*^−1^, the stress exponent *q* = 3.77 and the electrochemical parameters—were all calibrated and acquired through our research group’s prior high-temperature, high-pressure aqueous environment experiments.

## 3. Effect of Crack Characteristics on Crack Tip Mechanical and Creep Fields

### 3.1. Influence of Crack Position

#### 3.1.1. Effect of Creep on Mises Stress at the Crack Tip for Cracks at Different Sub-Interface Positions

**Figure 6:**
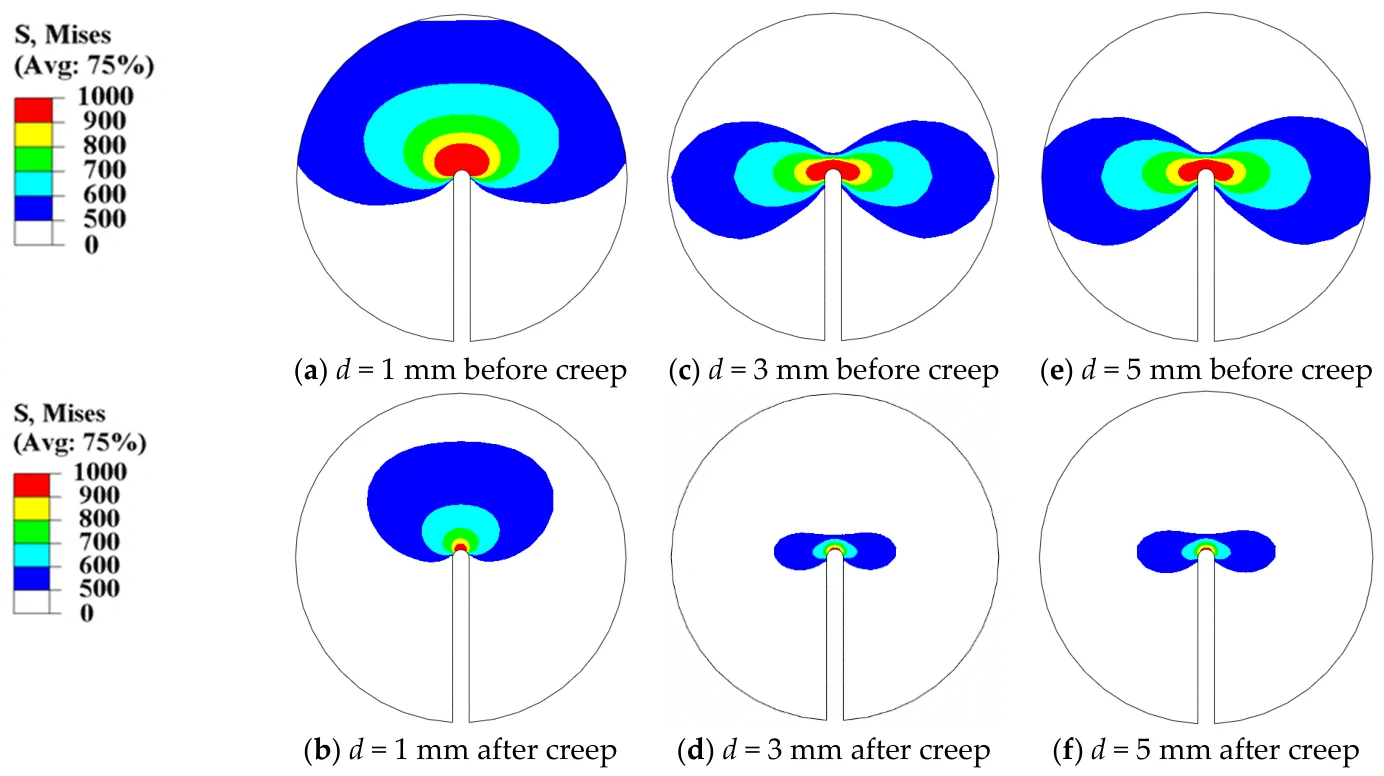
Contours of Mises stress distribution at the crack tip before and after creep under different crack positions at the weld interface (*a/W* = 0.5).

Figure 6 delineates the evolution of the Mises stress field surrounding cracks situated at varying distances from the sub-interface, comparing their states prior to and following the creep process. The crack length in Figure 6 corresponds to a relative depth of *a/W* = 0.5. As is evident from the contours, the stress field is characteristically localized, with peak magnitudes intensely concentrated in the immediate vicinity of the crack tip. Upon exposure to a 1000 h creep regimen, a significant stress relaxation phenomenon is observed across the board; specifically, for cracks positioned at sub-interface distances of *d* = 1 mm, *d* = 3 mm, and *d* = 5 mm, the formerly steep Mises stress gradients dissipate considerably, resulting in a more diffuse and gradual distribution profile. However, the persistence of elevated stress at *d* = 1 mm—despite global relaxation—reveals a critical crack-tip constraint effect: the severe mechanical mismatch between the softer 316L base metal and the harder Alloy 600 weld creates a geometrically constrained zone where plastic flow is restricted by the surrounding higher-strength material. This constraint sustains higher local stresses and impedes full stress relaxation.

A closer examination of the stress field morphology reveals distinct spatial characteristics contingent on crack location. When the crack initiates at *d* = 1 mm, the Mises stress distribution remains tightly constrained around the tip. Conversely, as the crack moves further afield to *d* = 3 mm and *d* = 5 mm, the stress field undergoes a morphological transformation, extending more prominently along the direction normal to the crack plane. Furthermore, an inverse relationship between distance and stress intensity at the tip is noted: the further the crack is removed from the sub-interface, the slightly higher the Mises stress at the tip becomes. This behavior is mechanistically attributed to the continuous increase in the yield strength of the weld metal with increasing distance from the interface, which alters the local load-bearing capacity and redistributes the stress concentration.

The morphological transition of the stress field—from tightly localized at *d* = 1 mm to extended normal to the crack plane at *d* = 3 mm and *d* = 5 mm—is governed by the stress redistribution mechanism associated with the spatially varying yield strength. As the crack moves away from the sub-interface, the increasing yield strength of the weld metal alters the local load-bearing capacity. The material ahead of the crack gradually shifts from a “soft-constrained” regime (near the interface) to a “stronger-but-more-homogeneous” regime, allowing the stress peak to redistribute over a broader region normal to the crack plane. This spatial realignment of the stress field directly modifies the Mode I opening traction, thereby influencing the driving force for SCC.

#### 3.1.2. Effect of Creep on PEEQ at the Crack Tip for Cracks at Different Sub-Interface Positions

**Figure 7:**
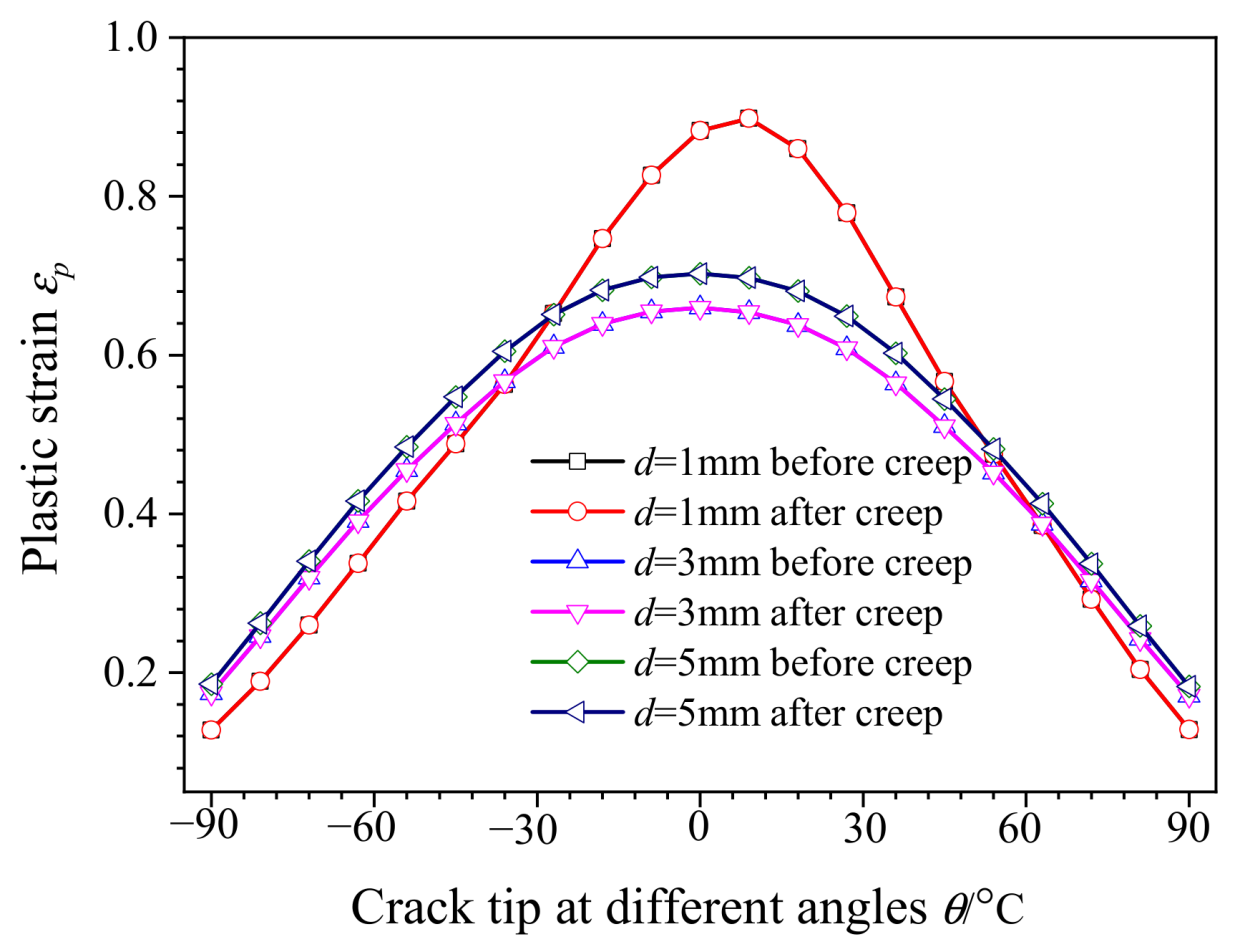
Circumferential PEEQ distribution before and after creep at the crack tip under different positions at the weld sub-interface.

**Figure 8:**
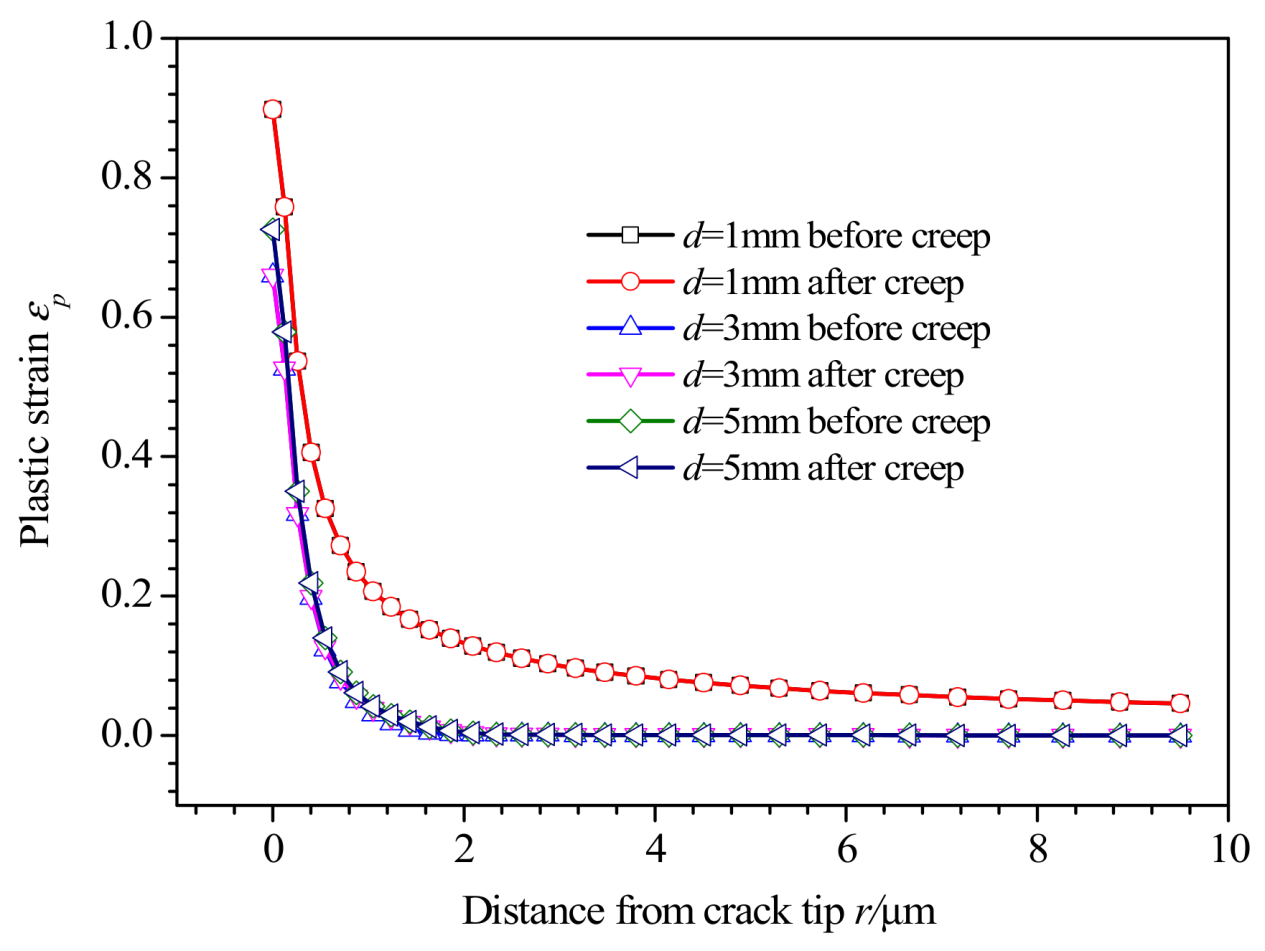
Horizontal directional PEEQ distribution before and after creep at the crack tip under different positions at the weld sub-interface.

The non-monotonic variation in equivalent plastic strain (PEEQ) with distance from the sub-interface (Figure 7 and Figure 8) is mechanistically rooted in the interaction between heterogeneity and crack-tip constraint. Figure 7 delineates the distribution of equivalent plastic strain (PEEQ) evaluated along Circumferential Path 1 at the crack tip, comparing the states preceding and subsequent to the creep exposure for cracks situated at varying distances from the weld sub-interface. A meticulous examination of the data reveals that the creep regimen, while inducing temporal evolution in strain magnitude, does not perturb the fundamental circumferential distribution pattern of plasticity. Notably, the PEEQ response exhibits a striking invariance with respect to the crack initiation site, underscoring a consistent mechanical behavior across the tested positions. The spatial localization of plastic deformation is distinctly characterized by a sharp concentration of peak PEEQ values within the angular sector spanning −30° to +30° relative to the crack tip axis, indicating that while the magnitude of plasticity evolves with time, the pattern of strain localization is intrinsically governed by the geometric and material constraints of the system rather than by time-dependent relaxation alone. Furthermore, the inherent mechanical property heterogeneity endemic to the dissimilar metal weld—specifically the juxtaposition of 316L stainless steel and Alloy 600 across the sub-interface—imparts a marked asymmetrical signature to the PEEQ profile along the circumferential trajectory.

Complementing this, Figure 8 elucidates the PEEQ distribution traced along Crack Propagation Path 2 for the same set of sub-interface distances. A clear positional dependence of plastic strain accumulation is observable: when the crack resides in closest proximity to the sub-interface (*d* = 1 mm), the material manifests the highest levels of equivalent plastic strain. As the crack location recedes to *d* = 3 mm, the PEEQ magnitude diminishes significantly, only to register a subsequent increase at the furthest evaluated distance (*d* = 5 mm). This non-monotonic variation is mechanistically rationalized by the continuously evolving mechanical properties of the weld deposit as a function of distance from the 316L-Alloy 600 interface, reflecting the gradient in material strength. Crucially, paralleling the observations from the circumferential analysis, the spatial profile of PEEQ along the direction of potential crack extension remains unaltered by the creep process, indicating that while the magnitude of damage evolves, the underlying strain localization pattern is intrinsically governed by the geometric and material constraints of the system.

### 3.2. Influence of Crack Length at the Weld Center

#### 3.2.1. Effect of Creep on Mises Stress at the Crack Tip for Central Weld Cracks of Different Lengths

**Figure 9:**
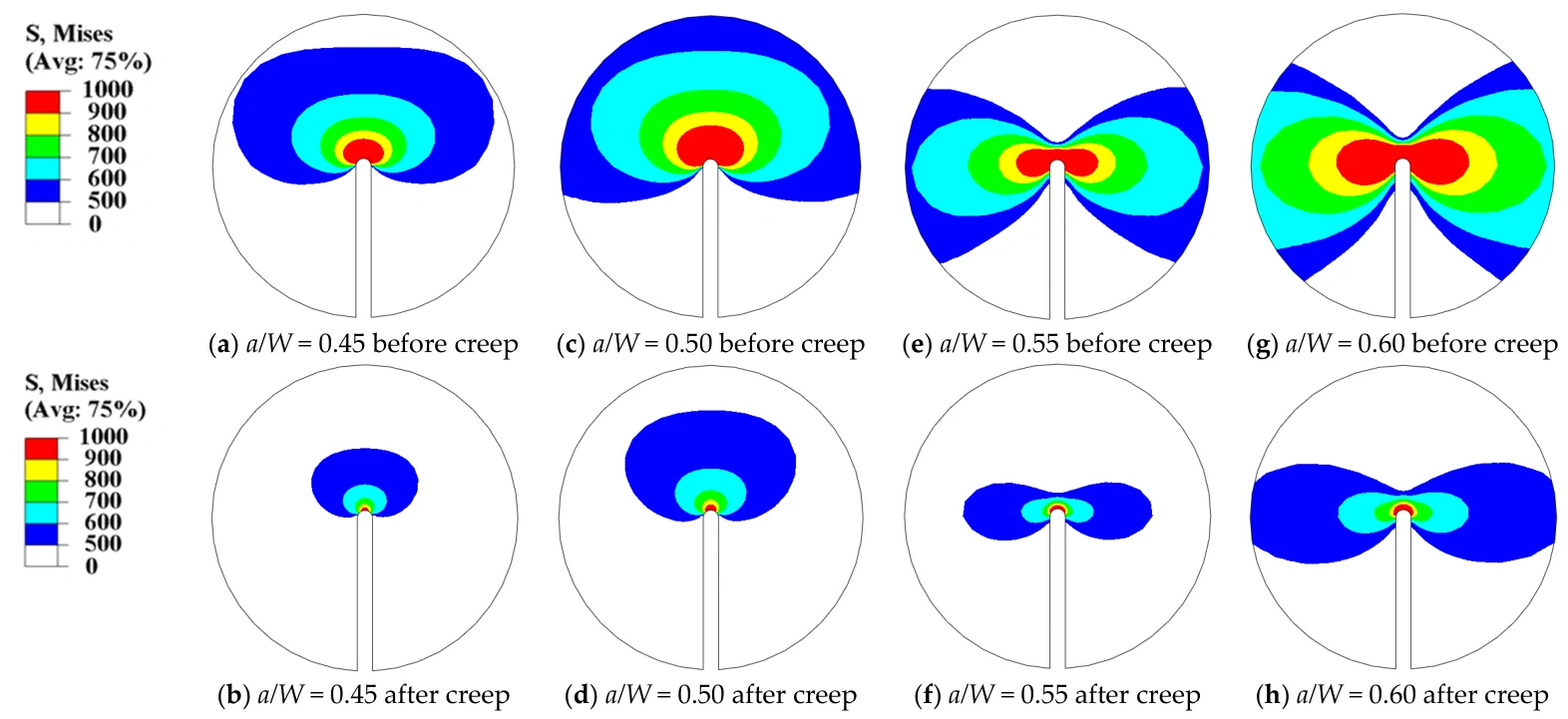
Contours of Mises stress distribution before and after creep at the crack tip for different crack lengths at the weld interface.

As delineated in Figure 9, the Mises stress profiles at the tips of cracks with varying lengths, prior to the onset of creep, register values that are orders of magnitude beyond the nominal yield strength of the material, manifesting as intensely localized concentrations within an exceedingly confined zone immediately adjacent to the crack apex. A distinct scaling effect is observable: the longer the pre-existing crack length, the more elevated the initial stress intensity prevailing at the tip. Subsequent to the 1000 h creep exposure, the stress field enveloping the crack tip undergoes a profound relaxation, characterized by a significant dissipation of the formerly steep stress gradients. Nevertheless, a residual hierarchy persists wherein the crack tip stress associated with longer flaws remains marginally superior to that of their shorter counterparts.

A comparative assessment of the Mises stress distribution morphologies—both prior to and following creep across the spectrum of crack lengths—reveals a distinct transition in the field characteristics. For cracks situated at the mid-section of the weld with relative depths of *a/W* = 0.45 and *a/W* = 0.50, the stress distribution remains tightly constrained and highly focused within the immediate crack tip vicinity. Conversely, as the crack length advances to *a/W* = 0.55 and *a/W* = 0.60, the Mises stress field undergoes a morphological shift, tending to extend more prominently along the direction normal to the crack plane. This specific redistribution profile effectively amplifies the magnitude of the normal tensile traction acting upon the prospective fracture plane. Consequently, this mechanistic insight indicates that, under identical remote loading conditions, longer cracks are endowed with a greater mechanical driving force, rendering them significantly more susceptible to environmentally assisted propagation, specifically SCC.

The scaling effect observed in Figure 9—where longer cracks (*a/W* = 0.55, 0.60) sustain higher residual stresses after creep—is a manifestation of crack-tip constraint loss with increasing flaw size. Shorter cracks (*a/W* = 0.45, 0.50) are embedded in a fully constrained, high-triaxiality stress field that promotes extensive creep relaxation.

#### 3.2.2. Effect of Creep on PEEQ at the Crack Tip for Central Weld Cracks of Different Lengths

**Figure 10:**
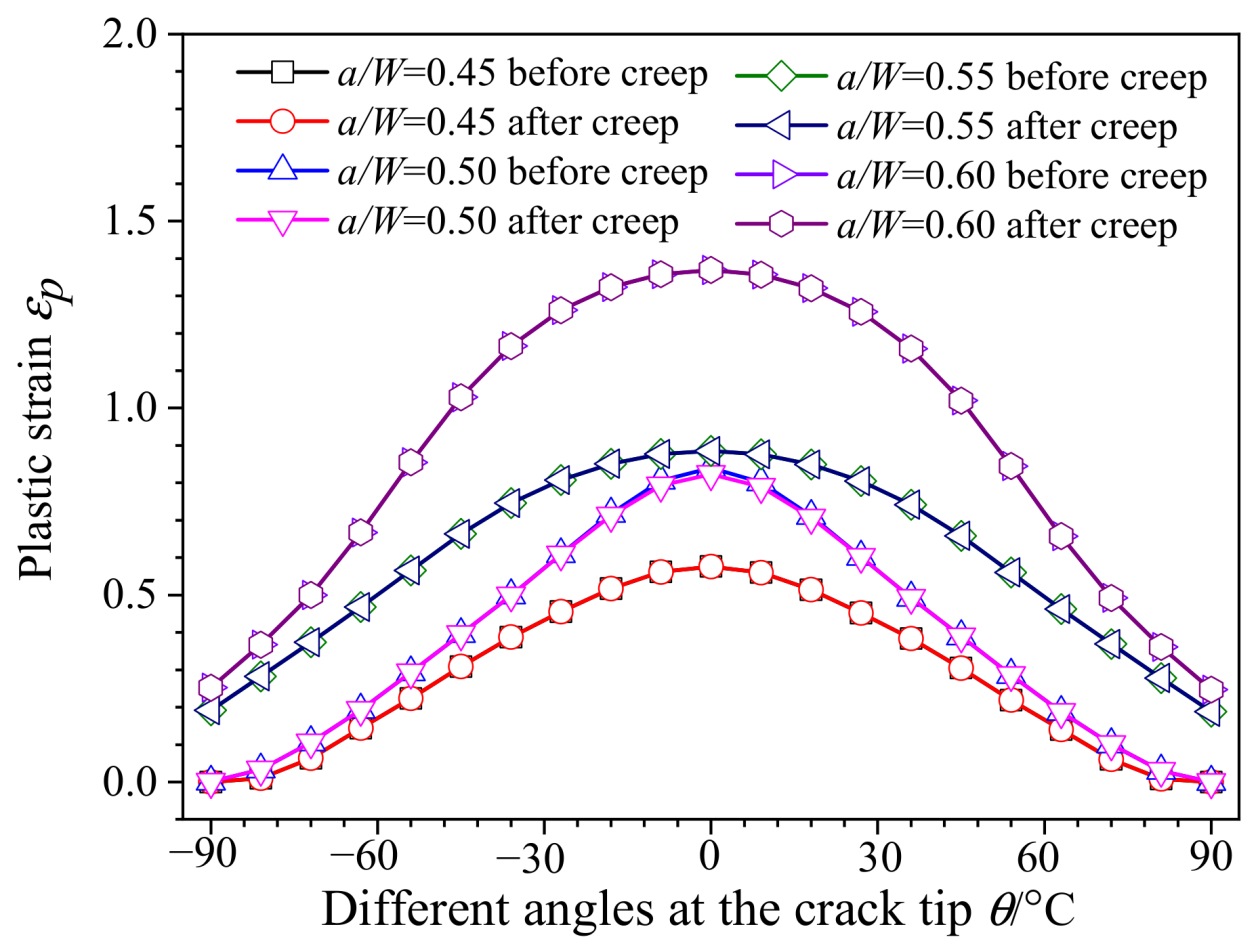
Circumferential PEEQ distribution before and after creep at the crack tip for different crack lengths at the weld interface.

Figure 10 delineates the distribution of equivalent plastic strain (PEEQ) along Circumferential Path 1 at the crack tip, evaluating cracks of varying lengths situated at the center of the weld interface both prior to and following the creep regime. A rigorous analysis confirms that irrespective of the crack propagation length, the accrued plastic strain field retains its intrinsic morphological signature; specifically, the overarching distribution pattern remains unperturbed by the 1000 h creep exposure. Further scrutiny reveals a length-dependent localization of peak plasticity: for intermediate crack depths of *a/W* = 0.45 and *a/W* = 0.50, the maximum PEEQ is tightly confined within the narrow angular sector spanning −30° to +30° relative to the crack tip axis. However, as the crack length transitions to deeper flaws (*a/W* = 0.55 and *a/W* = 0.60), the zone of intense plastic strain broadens significantly, extending across a wider angular range of −60°~60°. This pronounced augmentation of plastic strain within the sector aligned with the normal driving load serves as compelling micro-mechanical evidence, substantiating the accelerated propensity for SCC propagation in the presence of long cracks.

The broadening of the PEEQ angular sector from ±30° (*a/W* ≤ 0.50) to ±60° (*a/W* ≥ 0.55) (Figure 10) is a direct consequence of constraint degradation at larger crack depths. As the crack grows, the plastic zone size increases proportionally, reducing the shielding effect of the surrounding elastic material. The higher angular spread of plasticity means that a larger volume of material ahead of the crack is subjected to high plastic strains, which in turn accelerates oxide film rupture over a wider frontal area. This micro-mechanistic evidence strongly supports the macroscopically observed correlation between crack length and SCC propagation rate.

**Figure 11:**
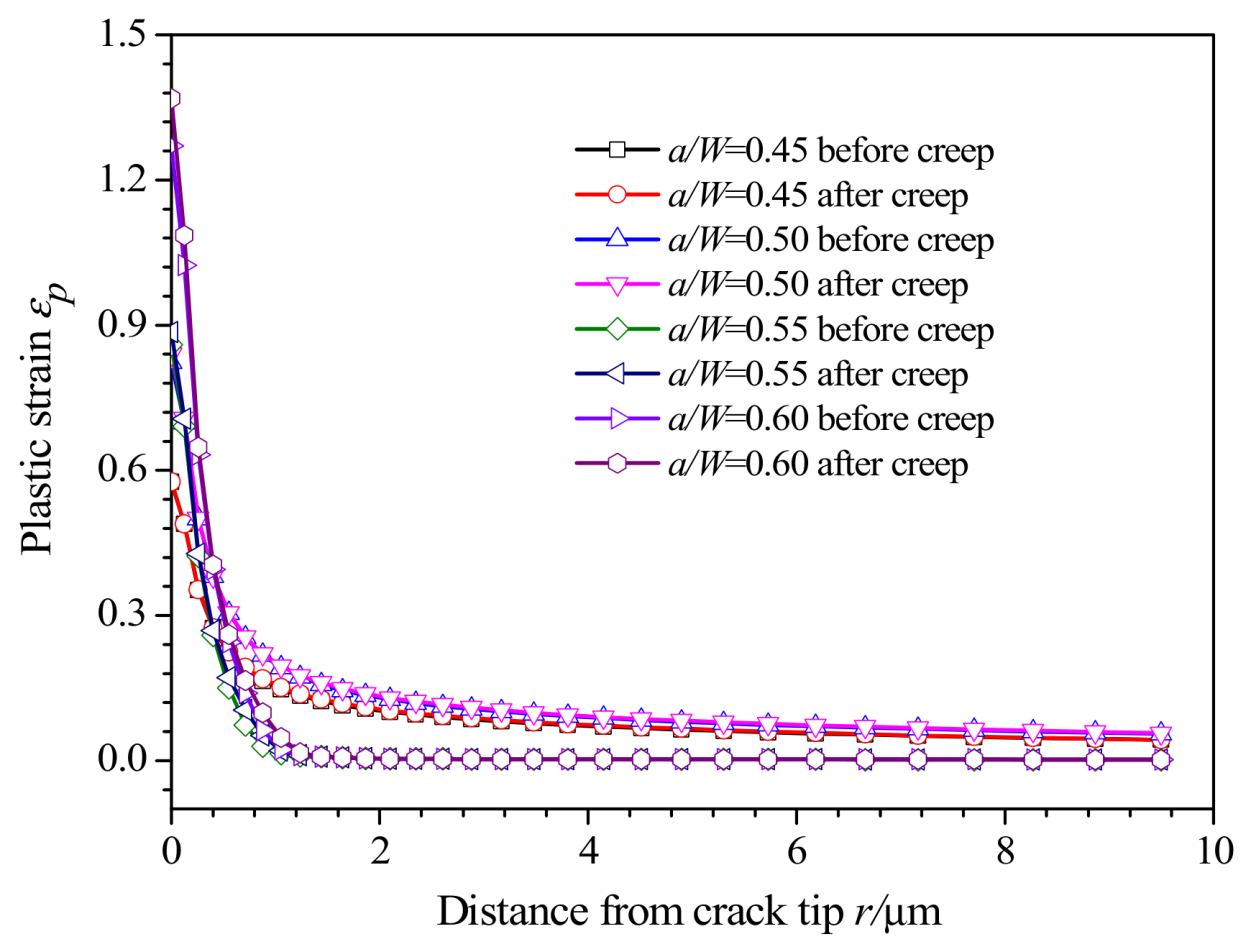
Horizontal directional PEEQ distribution before and after creep at the crack tip for different crack lengths at the weld interface.

Complementing this, Figure 11 elucidates the PEEQ distribution along Crack Propagation Path 2, traversing various locations within the crack tip region. The data unequivocally demonstrate a positive correlation between crack length and plastic damage accumulation: longer cracks induce substantially greater plastic deformation at the tip compared to their shorter counterparts. This disparity underscores the heightened energy dissipation and damage susceptibility associated with extended flaws. Concurrently, mirroring the observations along the circumferential path, the spatial profile of PEEQ traced along the direction of prospective crack extension exhibits remarkable stability, remaining invariant before and after the creep process. This consistency implies that while the magnitude of plasticity scales with crack length, the fundamental strain localization mechanism ahead of the crack front is inherently dictated by the prevailing mechanical constraints rather than time-dependent relaxation effects.

### 3.3. Quantitative Analysis of Crack Tip Creep Rate

#### 3.3.1. Effect on Crack Tip Creep When Cracks Are at Different Weld Positions

**Figure 12:**
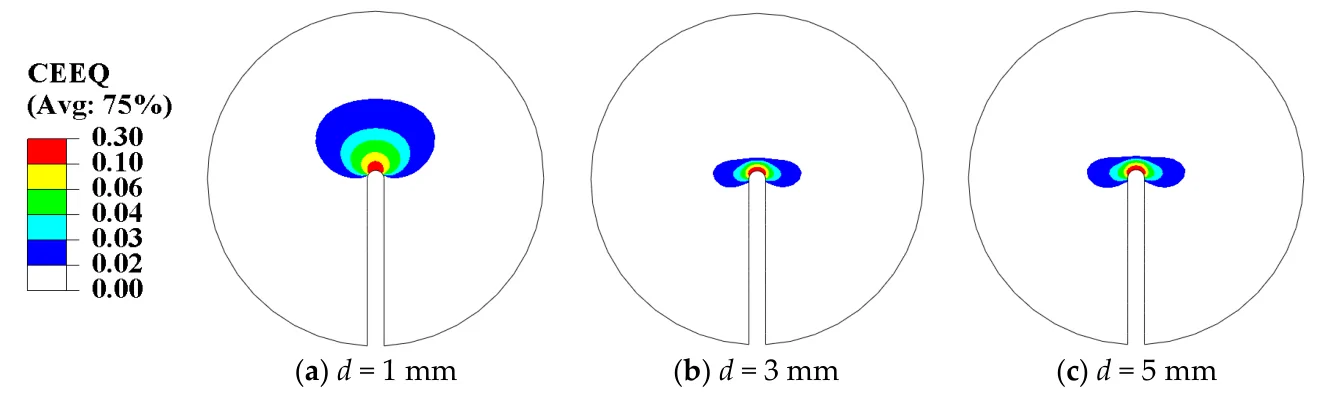
Contours of CEEQ distribution at the crack tip at the center of the weld interface (*a/W* = 0.5).

Figure 12 delineates the contour plots of Equivalent Creep Strain (CEEQ) accumulated after the 1000 h creep exposure, specifically contrasting cracks initiated at various distances from the sub-interface. The crack length in Figure 12 corresponds to a relative depth of *a/W* = 0.5. The spatial dependency of equivalent creep strain (CEEQ) in Figure 12 is governed by the coupled interaction between mechanical heterogeneity and creep deformation. The contours vividly illustrate a distinct spatial dependency of creep damage on positional heterogeneity. Most notably, the crack tip residing in closest proximity to the material discontinuity (*d* = 1 mm), sustains the most severe creep strain accumulation. This localized intensification is mechanistically attributed to the pronounced mechanical mismatch inherent to the dissimilar metal interface, which fosters a complex stress–strain state conducive to enhanced creep deformation.

As the crack initiation site recedes outward to *d* = 3 mm and *d* = 5 mm, the overriding influence of this interfacial mechanical heterogeneity gradually attenuates. Concomitantly, the CEEQ distribution profile evolves, progressively transitioning toward a more symmetric morphology characteristic of a more homogeneous material domain. Paradoxically, a comparative assessment between the *d* = 3 mm and *d* = 5 mmscenarios reveals an inverse trend: the locale further removed from the sub-interface (*d =* 5 mm) exhibits a quantitatively larger creep strain magnitude. This counter-intuitive elevation is fundamentally rooted in the evolving material properties of the weld deposit itself. Specifically, the progressive increase in yield strength encountered at greater distances from the 316L-Alloy 600 interface alters the local constraint factor, thereby elevating the steady-state creep rate and resultant strain in this region relative to the near-interface zone.

#### 3.3.2. Effect of Different Crack Lengths at the Middle of the Weld on Crack Tip Creep

**Figure 13:**
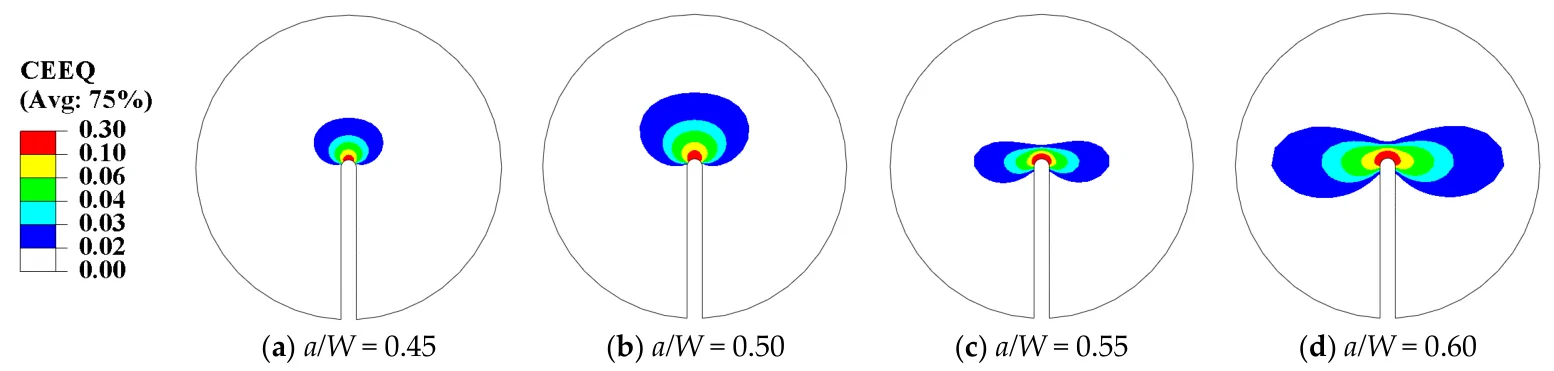
Contours of crack tip creep amount CEEQ distribution for different crack lengths at the middle weld interface.

Figure 13 delineates the Equivalent Creep Strain (CEEQ) contours localized at the crack tip, focusing on flaws of varying lengths situated at the central region of the nickel-based Alloy 600 weldment. The progressive escalation of CEEQ with crack length (Figure 13) is mechanistically linked to the constraint-mediated stress redistribution described in Section 3.2.1. The visualization vividly illustrates that the most intense creep damage is strictly confined to the immediate vicinity of the crack apex. A distinct scaling behavior is evident: as the crack propagation length advances, the magnitude of accumulated creep strain at the tip escalates correspondingly, reflecting a heightened susceptibility to time-dependent deformation for longer flaws.

A comparative interrogation of the CEEQ field morphology across the spectrum of crack lengths reveals a distinct transitional behavior. For intermediate crack depths at the weld midsection, specifically *a/W* = 0.45 and *a/W* = 0.50, the creep strain distribution remains tightly compacted, exhibiting a high degree of localization directly ahead of the crack tip. In stark contrast, as the relative crack depth transitions to deeper states (*a/W* = 0.55 and *a/W* = 0.60), the CEEQ footprint undergoes a morphological evolution, skewing preferentially toward the direction normal to the crack plane. This reorientation of the creep strain field serves to augment the magnitude of the normal tensile creep strain component acting upon the prospective fracture plane. Mechanistically, this distribution profile effectively amplifies the creep driving force, thereby elucidating why, under identical service loading conditions, longer cracks possess a markedly lower resistance to sustained crack extension and are thus predisposed to more facile propagation.

#### 3.3.3. Influence of Different Weld Positions and Crack Lengths on the Crack Tip Creep Rate

**Figure 14:**
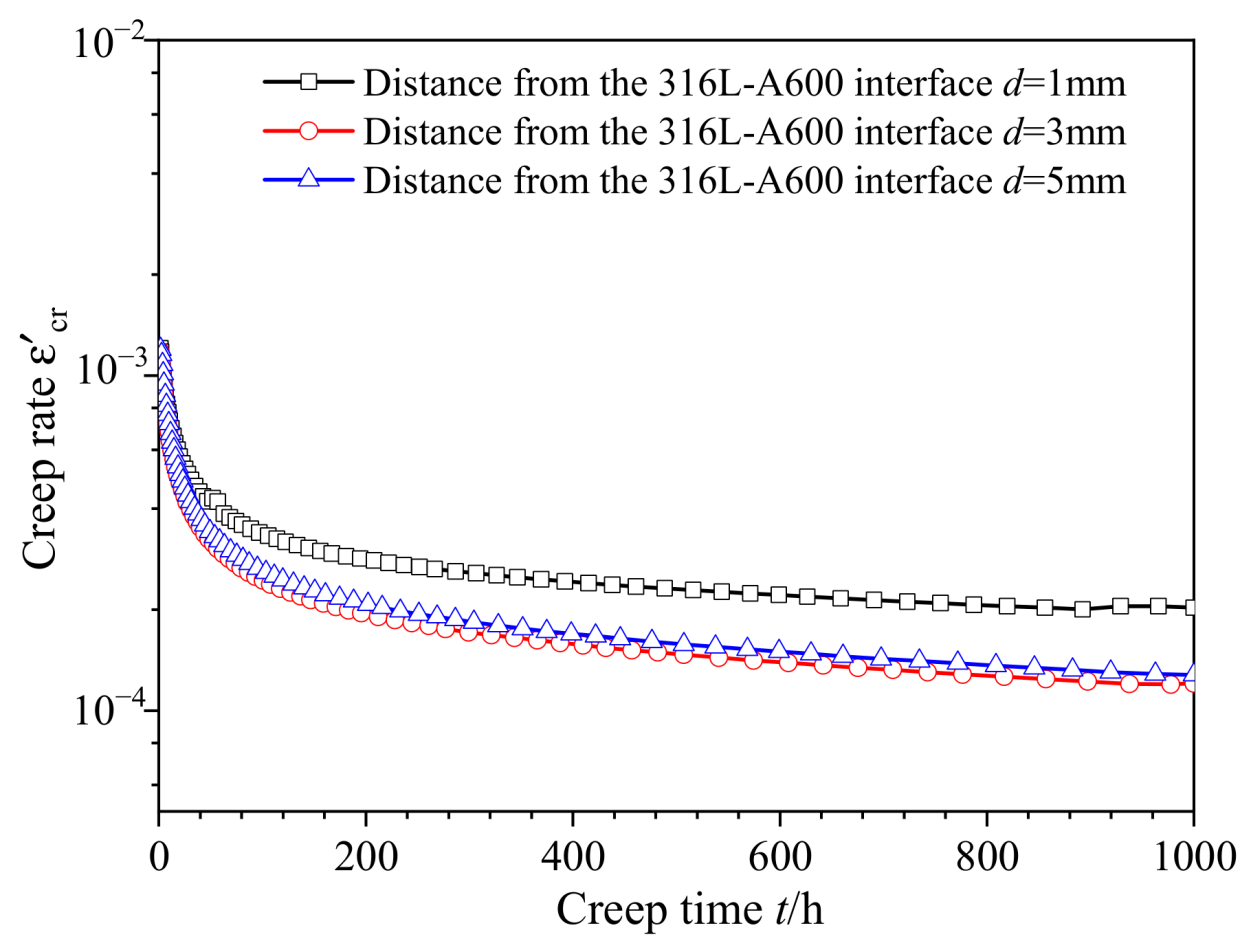
Effect of different distances from the weld on the crack tip creep rate.

Figure 14 elucidates the nuanced relationship between the crack tip creep rate and the spatial proximity to the weld interface. The data conspicuously demonstrate that when the crack is situated at a standoff distance of merely *d* = 1 mm from the dissimilar metal junction—specifically the nickel-based Alloy 600 to stainless steel 316L interface—it endures a markedly elevated creep rate. This acute acceleration is mechanistically ascribed to the intense mechanical heterogeneity endemic to the sub-interface region, where the stark property mismatch between the base and weld metals engenders a complex, high-magnitude stress state conducive to rapid time-dependent deformation.

Conversely, as the distance parameter increases to 3 mm and subsequently to 5 mm, the overriding influence of this interfacial heterogeneity progressively attenuates. In this regime, the material behavior is increasingly governed by the intrinsic property gradient of the weld deposit itself. Notably, the concomitant continuous evolution in yield strength—which experiences a marginal yet discernible increment at locations further removed from the interface—paradoxically facilitates a slight amplification in the crack tip creep rate. This subtle upturn underscores the intricate interplay between material strengthening and creep response within the transitioning microstructure of the weldment. It should be noted that while the steady-state creep rate is attained at *t* ≈ 200 h, the overall simulation duration of 1000 h was chosen to guarantee full abatement of transient effects and to generate a measurable level of creep strain accumulation for reliable post-processing and visualization.

**Figure 15:**
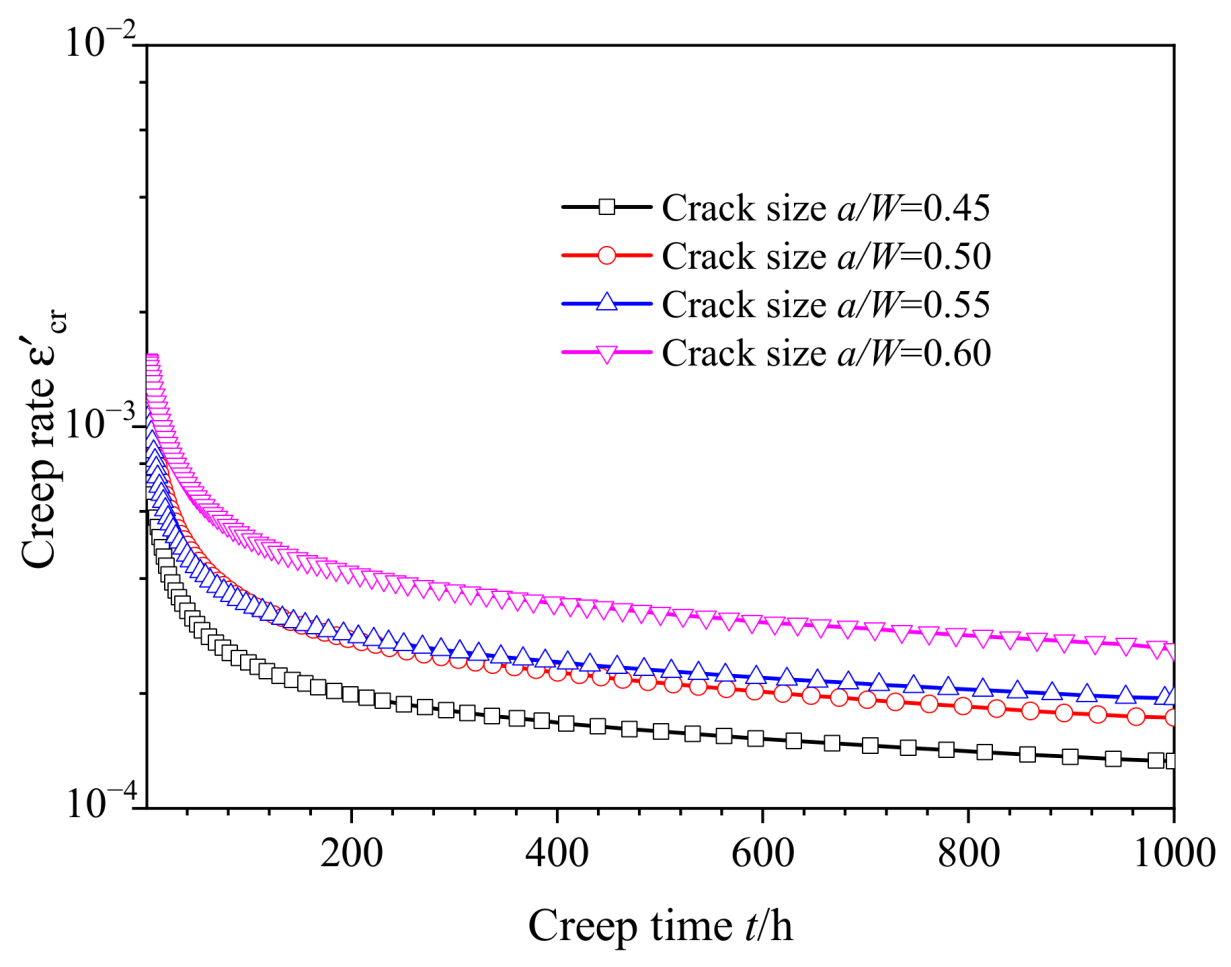
Effect of different crack lengths at the weld on the crack tip creep rate.

Figure 15 delineates the functional relationship between the crack tip creep rate and the relative crack depth emanating from the weld centerline. A conspicuous trend is immediately apparent: for the shortest crack configuration examined (*a/W* = 0.45), the crack tip sustains its minimum creep rate, indicative of a lower susceptibility to time-dependent deformation in the early stages of flaw growth. As the crack propagation length undergoes a progressive elongation, the creep rate within the crack tip region ascends correspondingly, achieving progressively higher values during the steady-state phase.

Figure 14 and Figure 15 collectively demonstrate that the crack tip creep rate is governed by two competing mechanisms: (i) mechanical mismatch-induced constraint near the sub-interface, which dominates at small distances (*d* = 1 mm) and elevates creep rates; and (ii) yield—strength—mediated flow stress elevation, which becomes predominant at larger distances (*d* ≥ 3 mm) and with increasing crack length. The attainment of a steady-state plateau at *t* ≈ 200 h (Figure 15) signifies the full abatement of transient creep effects, confirming that the reported SCC rates are representative of long-term service conditions.

The synthesis of these observations provides a unified mechanistic framework: mechanical heterogeneity modifies crack-tip constraint, which in turn dictates the stress redistribution under creep, and the resulting stress state—combined with the local yield strength gradient—determines the steady-state creep rate and, ultimately, the SCC propagation kinetics. This framework substantiates the quantitative predictions presented in Section 3.4 and validates the superiority of the heterogeneous gradient model over homogeneous-material assumptions.

### 3.4. SCC Growth Rate Prediction and Analysis Based on Continuous Gradient Field

**Figure 16:**
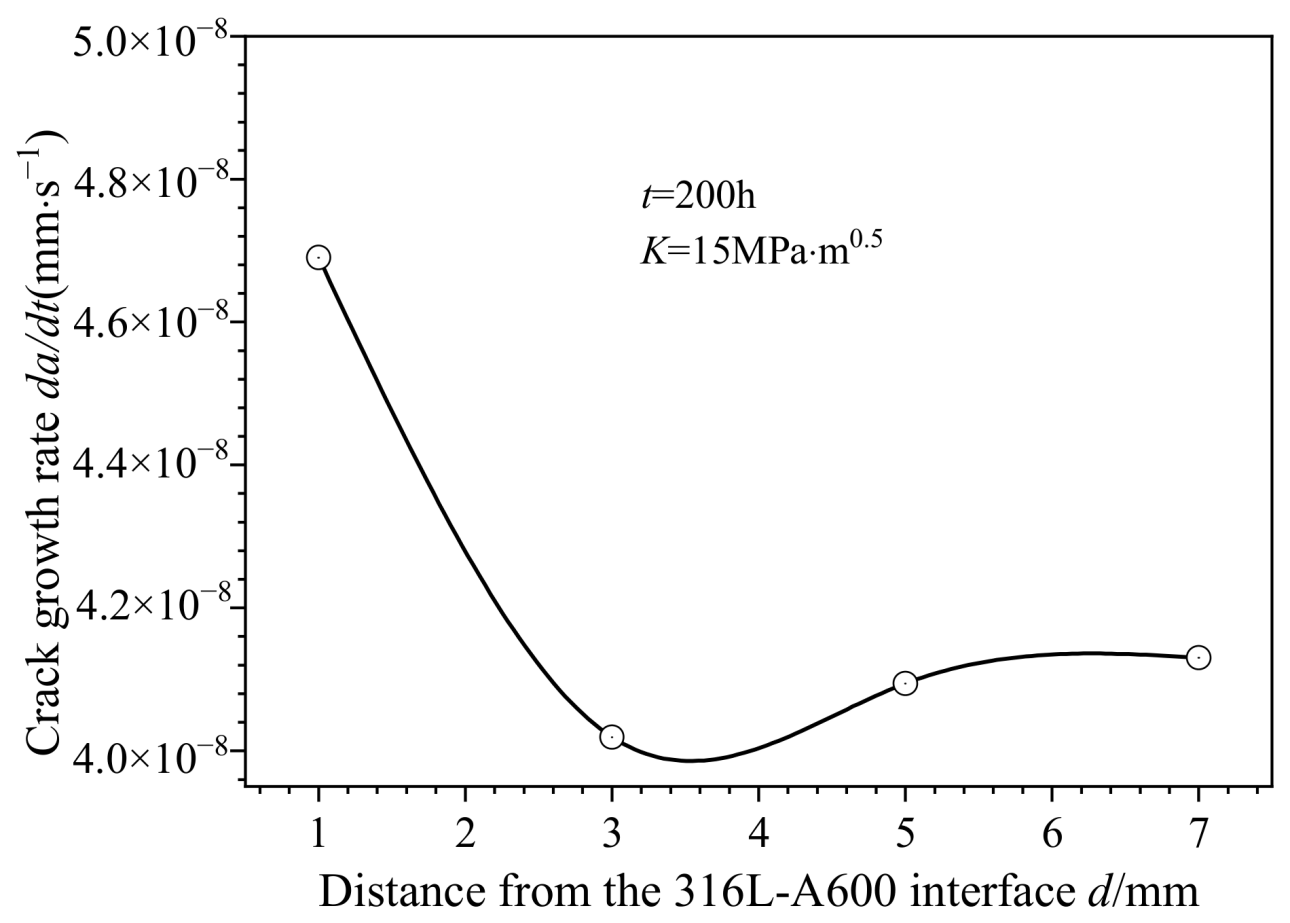
Effect of Different Distances from the Weld Joint on the SCC Growth Rate.

By substituting the numerically derived steady-state crack tip creep rates—calculated for various crack positions (including an extended prediction at *d* = 7 mm) and propagation lengths—into the SCC propagation model (Equation (2)), Figure 16 shows the predicted SCC growth rate as a function of distance from the weld sub-interface. The extension to 7 mm captures the full non-monotonic trend, confirming a secondary rate increase at larger distances driven by the continuous elevation of yield strength away from the interface. A critical dependency emerges from the data: when the crack is situated at a standoff distance of *d* = 1 mm from the dissimilar metal junction (i.e., the interface between the nickel-based Alloy 600 weld and the 316L stainless steel base material), the SCC propagation rate peaks sharply. This acceleration is mechanistically rooted in the intense mechanical heterogeneity endemic to the sub-interface region, where the stark property dichotomy between the base and weld metals creates a high-energy driving force for environmentally assisted cracking. As the positional parameter extends outward to 3 mm, 5 mm, and 7 mm, the overriding influence of this interfacial heterogeneity progressively attenuates. Paradoxically, the concomitant continuous evolution in yield strength across the weld deposit—characterized by a marginal yet discernible increment at locales further removed from the interface—facilitates a subtle but measurable amplification in the SCC propagation rate despite the reduced heterogeneity.

**Figure 17:**
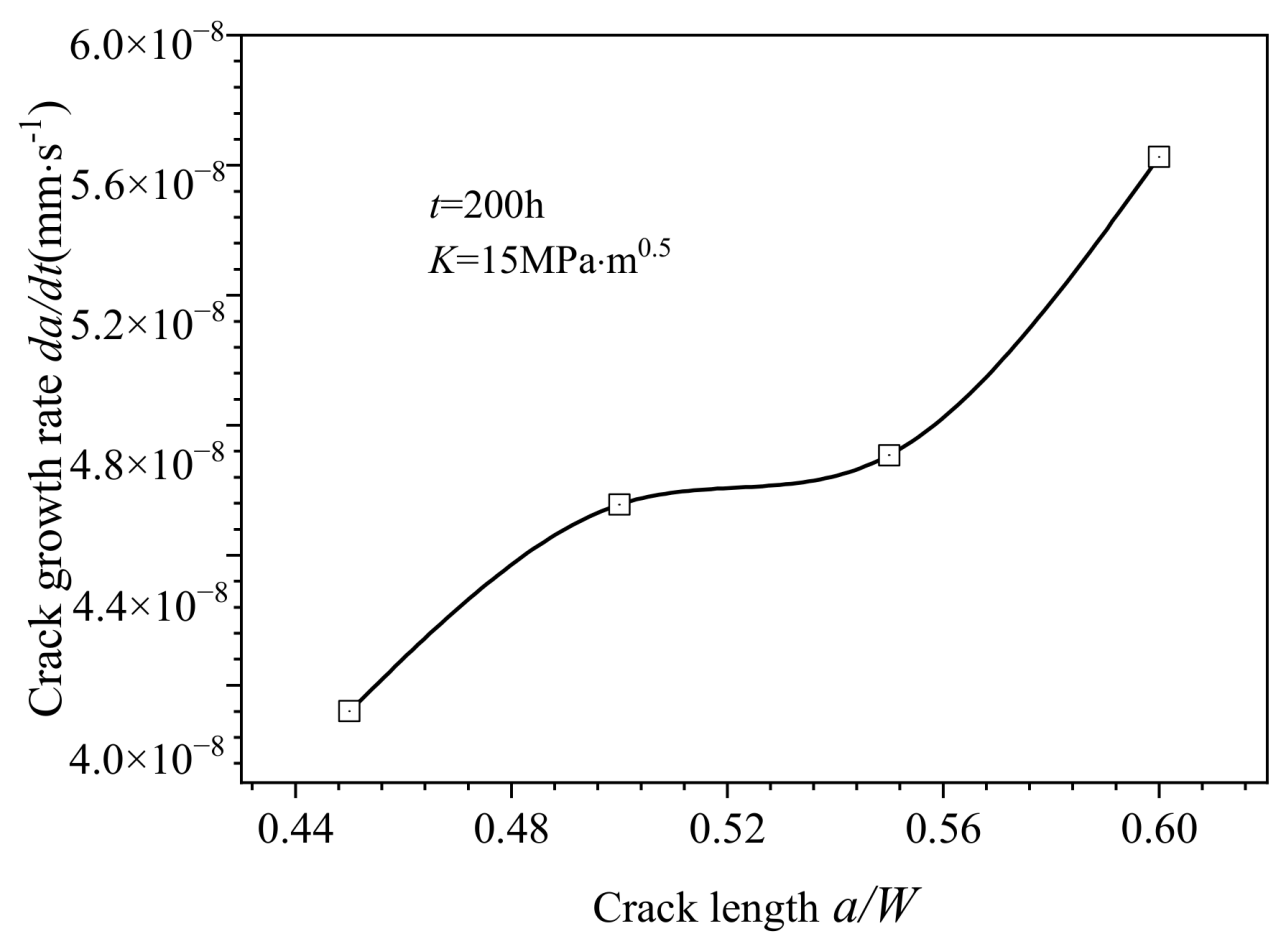
Influence of Different Crack lengths at the Weld Center on the SCC Growth Rate.

Complementing this positional analysis, Figure 17 elucidates the effect of varying crack depths at the weld centerline on the SCC propagation kinetics. The curve reveals a monotonic, albeit non-linear, escalation in crack growth velocity with increasing flaw size. Consistent with prior observations, the shortest crack length evaluated (*a/W* = 0.45) corresponds to the minimal SCC propagation rate, reflecting a lower driving force for damage accumulation. As the crack advances through the intermediate stages (*a/W* = 0.50 to *a/W* = 0.55), the rate of increase in SCC propagation remains modest. This transitional behavior is mechanistically attributed to the previously identified morphological shift in the crack tip creep strain field—evolving from a tightly concentrated zone to a more broadly dispersed distribution normal to the crack plane, which modulates the local tensile driving force. Ultimately, upon reaching the deepest evaluated flaw size (*a/W* = 0.60), the SCC propagation rate culminates at its absolute maximum, underscoring the criticality of crack length as a governing parameter in long-term structural integrity assessments.

From an engineering perspective, although the absolute differences in SCC growth rates among these locations may appear modest, they carry significant implications for the long-term integrity of nuclear components. Over the typical design lifetime of a nuclear power plant (40–60 years), even a small sustained elevation in growth rate can accumulate into a noticeable difference in crack depth. For instance, the peak rate at *d* = 1 mm is substantially higher than at other positions; this implies that cracks initiating within 1 mm of the sub-interface will propagate faster, potentially reducing the time to reach a critical flaw size and necessitating more conservative inspection intervals. On the other hand, the slight increase in growth rate at larger distances (*d* = 5–7 mm) due to the rising yield strength suggests that the weld metal away from the interface is not immune to accelerated SCC. Conventional life prediction models that assume homogeneous properties would fail to capture these location-specific variations, likely leading to non-conservative estimates if a crack happens to initiate near the sub-interface. Therefore, the heterogeneous model presented here provides a more reliable basis for residual life assessment and risk-informed maintenance planning of DMWJs in nuclear primary circuits.

## 4. Conclusions

(1) Due to severe mechanical mismatch near the 316L–Alloy 600 sub-interface (*d* = 1 mm), the crack tip sustains the highest Mises stress and creep strain after 1000 h of creep, resulting in a peak SCC propagation rate that is substantially higher than at other locations (*d* = 3 mm, 5 mm). As the distance from the interface increases, the interfacial heterogeneity effect weakens, but the continuous increase in yield strength causes a slight secondary rise in creep strain and SCC rate at larger distances (*d* = 5–7 mm). Moreover, increasing the crack length at the weld center (*a/W* from 0.45 to 0.60) expands the plastic strain zone and monotonically elevates the steady-state creep rate and SCC growth rate. Creep does not change the circumferential distribution pattern of PEEQ, but it significantly amplifies the magnitude of PEEQ accumulation.
(2) The study reveals that the coupled interaction between mechanical heterogeneity and creep deformation governs SCC kinetics. Near the sub-interface, high crack-tip constraint imposed by the property mismatch sustains elevated stresses and accelerates creep; away from the interface, the increasing yield strength raises the local flow stress, which—under the power-law creep constitutive relation—exponentially amplifies the creep rate. These mechanisms, together with crack-length-induced constraint loss and stress redistribution, explain the observed non-monotonic and monotonic trends. This work therefore provides a deeper mechanistic understanding of environment-assisted cracking in graded material systems.
(3) From a structural integrity perspective, the location-dependent and length-dependent variations in SCC growth rate carry significant consequences for the long-term operation of nuclear power plants. Over a typical 40–60-year design life, even a small sustained elevation in growth rate near the sub-interface can accumulate into a considerable crack extension, potentially reducing the time to reach a critical flaw size. Consequently, cracks initiating within ~1 mm of the dissimilar metal interface demand more conservative safety margins and possibly more frequent in-service inspections. The heterogeneous gradient model presented here offers a more reliable basis for residual life assessment, risk-informed maintenance planning, and the optimization of inspection strategies for nuclear primary circuit components.

While this study provides significant insights into the influence of mechanical heterogeneity on SCC propagation, it is important to acknowledge certain limitations of the current numerical framework. The present model utilizes predefined crack configurations (fixed lengths and positions) to systematically evaluate the crack tip mechanical fields. Consequently, the autonomous evolution of the crack path (spontaneous extension) and potential crack deflection phenomena, frequently observed in complex DMWJs, are not captured. Future work will focus on addressing these limitations by implementing damage mechanics-based approaches or the extended finite element method (XFEM) to simulate crack advance.

## Data Availability

The original contributions presented in this study are included in the article. Further inquiries can be directed to the corresponding author.
